# Factors Affecting the Formation of 2:1 Host:Guest Inclusion Complexes of 2-[(R-Phenyl)amine]-1,4-naphthalenediones (PAN) in β- and γ-Cyclodextrins

**DOI:** 10.3390/molecules21111568

**Published:** 2016-11-18

**Authors:** Christopher K. Jankowski, Christine Lamouroux, Manuel Jiménez-Estrada, Sebastien Arseneau, Brian D. Wagner

**Affiliations:** 1 Département de Chimie et Biochimie, Université de Moncton, Moncton, NB E1A 3E9, Canada; krzysztof.jankowski@umoncton.ca (C.K.J.); christine.lamouroux@areva.com (C.L.); sebastien.arseneau@hotmail.com (S.A.); 2 Instituto de Química, Universidad Nacional Autónoma de México, Ciudad Universitaria, México D.F. 04510, Mexico; manueljemex@gmail.com; 3 Department of Chemistry, University of Prince Edward Island, Charlottetown, PEI C1A 4P3, Canada

**Keywords:** cyclodextrins, cyclodextrin inclusion complexes, ESI-MS, molecular modeling, host-guest inclusion, host-guest stoichiometry

## Abstract

The molecular hosts cyclodextrins form inclusion complexes with a wide variety of guests, resulting in complexes with various host:guest stoichiometries. In the case of a series of 19 1,4-naphthoquinolines as guests with either β- or γ-cyclodextrin studied using electrospray mass spectroscopy, in most cases only 1:1 complexes were observed, with 2:1 host:guest complexes observed in just 6 out of 38 host:guest combinations. It is shown that these higher-order complexes were observed only in the case of small (or no) electronically withdrawing substituents, and were much less likely in the case of the larger γ-cyclodextrin host. The size and electronic properties of the substituents involved shows that both steric and electronic factors must be taken into account in predicting which cyclodextrin host:guest stoichiometries will be stable enough to form (or once formed, be robust enough to be observed in the ESI-MS experiments). It is clear that the prediction of host-guest stoichiometry for a specific host-guest pair is complicated, and involves a subtle interplay of both electronic and steric factors. However, there are definite trends, which can be used to help predict host:guest stoichiometry for a given host-guest pair.

## 1. Introduction

Cyclodextrins (CDs), the cyclic oligomers of glucopyranose, are by far the most commonly used organic host compounds for the inclusion of organic guest molecules [1,2,3]. The inclusion of guest molecules into cyclodextrin host cavities results in significant changes to the guest properties, including solubility [4], spectroscopic properties [5] and stability [6]. These effects of cyclodextrin inclusion have been utilized in a wide variety of research and commercial applications [1,2,3], including in chromatography [7], trace analysis [8], drug delivery [9], guest stabilization [6], food science [6] and industrial applications [10].

However, it remains challenging to predict the stoichiometry of cyclodextrin host:guest inclusion complexes. In a broad sense, consideration of the size of the guest will to some extent help to predict this stoichiometry, as guests that are small relative to the native cyclodextrin host cavity diameter will tend to form 1:2 CD:guest complexes [11]; those that are similar in size to the CD cavity will form 1:1 complexes [12]; and those that are significantly larger (or in particular significantly longer) than the host cavity will tend to form 2:1 cyclodextrin:guest complexes [13,14]. However, small changes in guest shape can have a significant effect on the observed stoichiometry. In addition, higher order complexes have also been reported, including 2:2 [13] and even 2:3 [14], and in the solid state, even higher order cyclodextrin complexes have been reported, involving 3 CD hosts [15], and even 4:3 [16] and 4:5 [17] overall CD:guest stoichiometries! Moreover, in some cases, a mixture of complexes with different stoichiometries can be observed for a single host-guest pair [13,18,19], resulting in non-integer average stoichiometries (e.g., 1.3:1) [19]. In this paper, we report an example of a cyclodextrin:guest system in which varying the specific substituent on the guest, in this case 1,4-naphthoquinoline, results in a change in stoichiometry from 1:1 to 2:1. These results will be analyzed and discussed with the aim of furthering the current understanding of the host:guest inclusion phenomenon, and the prediction of host:guest stoichiometry, for cyclodextrin hosts.

**Figure 1:**
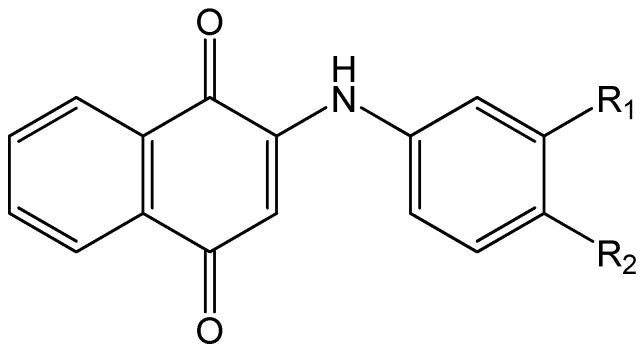
General structure of the 2-[(R-phenyl)amine]-1,4-naphthalenedione guests of interest. The list of the 19 specific guests studied and their R_1_ and R_2_ substituents is given in Table 1.

The host:guest inclusion complexes of 1,4-naphthoquinolines have been extensively studied because of their applications in medicine and biology [20]. The *p*-quinone system is quite popular as a part of many natural products [18,21] Furthermore, the electrochemistry of these and related systems has been well studied [22,23,24,25,26]. For example, this system allows for electron transfer to occur in bacteria [20,22], mitochondria [20,23] and chloroplasts [20,24]. In addition, 1,4-naphthoquinolines have found many practical applications, including as fungicides [27] and antimalarial agents [28]. Recently, some newer 1,4-quinone structures have been prepared and studied by one of our co-authors and his co-workers, most interestingly those having an N-mono substituted amine group in position 2 of the quinone ring [29,30,31]. These compounds, in particular the 2-[(R-phenyl)amine]-1,4-naphthoquinone (PAN) (R = substituent) (**1**) and its eighteen derivatives were synthesised by a member of our group [29,30] and studied as dyes for their effects on various systems [29,30,31]. These syntheses were designed in order to vary the size of the substituent on the benzene ring and to assure different electronic effects of these substituents (Figure 1).

We decided to undertake further study of this family of compounds using a new approach, namely supramolecular host-guest inclusion, whereby the PAN derivatives will be the guest molecules and enter the internal cavity of cyclodextrin (CD) hosts, in particular β- and γ-CD (α-CD was not used due to its small cavity size relative to PAN). The transport of PAN encapsulated in the CD hosts in biological systems is of significant interest. In their recent studies Gomez-Sandoval [29], Wen [32] and Kobetic [33] studied the host-guest inclusion and the stoichiometry of the resulting complexes of various biologically active compounds. Interestingly, it was found that such complexes often involved a 2:1 host:guest ratio, for example with the antiviral bioactive agent acyclovir [34]. This observation was subsequently related to central issues in molecular recognition and inclusion phenomenon, namely the size-fit and geometric complementarity factors involved in determining the inclusion complex stoichiometry for a specific host-guest pair [35].

For this investigation the most important technique for studying the inclusion phenomena was ESI mass spectroscopy [36,37,38], which is a relatively straightforward and rapid technique, which directly and rapidly determines the CD:guest stoichiometry (from the observed mass of the complexes), without using complex analysis and fitting techniques, as are required for example for microcalorimetry or fluorescence spectroscopy. We [39] and others [40] have recently studied, using ESI-MS, the use of various CD hosts, of different sizes, in the context of the transport of drugs through the digestive system [39] or to encapsulate drugs such as analgesics [40]. We have also previously used ESI mass spectroscopy, in combination with fluorescence spectroscopy, to study and characterize the binding of the fluorescent probe Nile Red in various cyclodextrins [41]. In this case the formation in solution of CD: guest complexes at 1:1 or 2:1 could easily be detected. In parallel, we use molecular modelling, which has been shown to produce quite reliable data for the stability of such complexes [32,39,41,42,43,44], and also allows for the calculation of the energies of potential 1:1 or 2:1 host:guest complexes.

The relative abundance of complexes with varying stoichiometries in the ESI-MS experiments is directly related to the energy and thus stability of these complexes. Wen et al. [32] calculated the energies of such complexes in the case of the malarial drug chinchonine using molecular modeling, to explain their ESI-MS results. We will use a similar approach in this work; however, we were also fascinated by the observed persistence of 2:1 complexes in the case of certain derivative guests, and their rather unusual behaviour under ESI conditions, and the reasons why some substituted PAN derivatives showed 2:1 complexation in the ESI-MS results, but the majority did not. In order to follow this trend we used in our ESI experiments the condition of excess of cyclodextrin in order to assure the formation and observation of only the most stable multi-cyclodextrin adduct ions. When the Wen group used protected CDs [32], many unexpected reactions took place, for example dealkylation or sharing of guests between cyclodextrin hosts. For this reason we preferred, as suggested by Kobetic [33], using the “native” CDs of differing size, namely β- and γ-CD, which have an appropriate cavity size for these guests. Furthermore, we decided to perform molecular modelling studies of the 1:1 and 2:1 host:guest complexes, with the goal of observing some significant differences justifying the stability of some of these complexes and the absence of others. Overall, our interest in unusual, uncommon or simply strange structures, proposed for ion structures observed under ESI conditions, was undertaken to rationalise the observed complexes using the thermodynamic calculations via molecular modelling (MM+ in particular as a first step) to develop a hypothesis for their formation and in particular to explain the unusual stability in solution of some of these complexes. 

We present herein the comparison of the data obtained from the mass spectrometry of the 19 PAN dyes (shown in Figure 1 and Table 1) and two CDs, namely β- and γ-CD, and the corresponding data of calculated energies for 1:1 and 2:1 complexes obtained via molecular modelling. The experimental and calculated data and results will be discussed in the following sections. Surprisingly, the 1:1 complexes between PAN and CD are not easily formed under the standard experimental conditions for ESI/PI (methanol, drop of formic acid, optimized ionization energy to observed the complex ions), and were only observed at a relatively high excess of CD. As will be discussed below, under such excess CD conditions, 1:1 and in the case of some host-guest pairs 2:1 host:guest complexes were observed.

## 2. Results

### 2.1. Electrospray Ionization Mass Spectroscopy (ESI-MS) Studies

**Figure 2:**
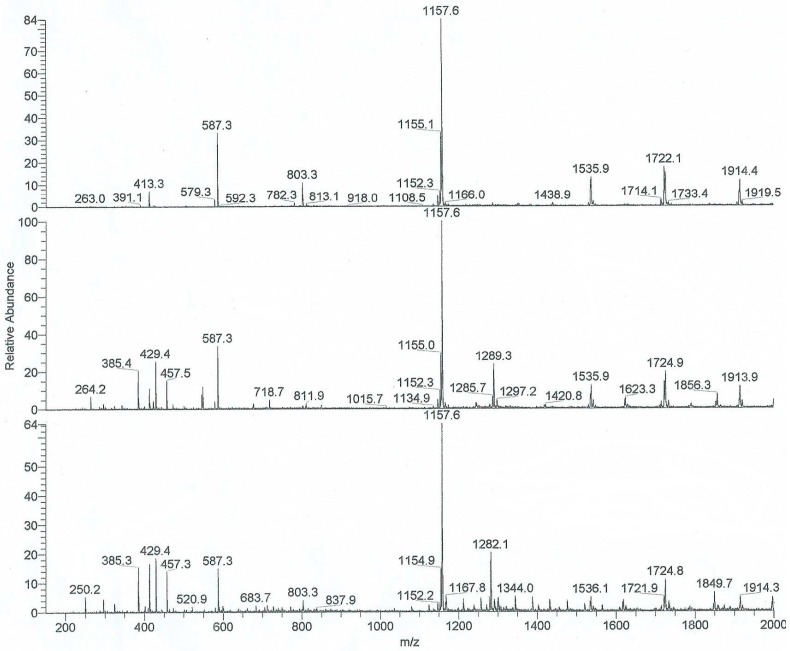
ESI-MS spectra of 2-[(*p*-methyl-phenyl)amine]-1,4-naphthalenedione and β-CD. **Upper** **spectrum**: β-CD control: MNa^+^ ion at *m*/*z* 1157.6; **Middle spectrum**: β-CD (2 × 10^−5^ M) complexed with PAN **10** (2 × 10^−6^ M), ratio 10:1; 2 β-CD + PAN **10** ion at *m*/*z* 1289.2 (MNa_2_)^2+^; PAN **10** MH^+^ ion at *m*/*z* 264.2; β-CD MNa^+^ ion at *m*/*z* 1157.6; **Bottom Spectrum**: β-CD (2 × 10^−5^ M) complex with PAN 1 (2 × 10^−6^ M), ratio 10:1; 2 β-CD + PAN 1 ion at *m*/*z* 1282.1 (MNa_2_)^2+^; PAN 1 MH^+^ ion at *m*/*z* 250.2; β-CD MNa^+^ ion at *m*/*z* 1157.6. When the MS/MS experiments were performed on (CD_2_MNa_2_)^2+^ ion formation of daughter ions at (CDMNa)^+^ and (CD_2_MH)^+^ were not observed; the only daughter ions recorded were (CDNa)^+^ and MH^+^.

ESI-MS experiments were performed for all 19 guests in both β- and γ-CD; the results of these experiments in terms of the observation of host:guest inclusion complexes, and their stoichiometry, are presented in Table 1. Representative ESI-MS spectra for the host β-CD itself, as well as the two specific host-guest pairs involving PAN derivative **10** (2-[(*p*-methyl-phenyl)amine]-1,4-naphthalenedione) or the parent PAN **1** both in the presence of β-CD are shown in Figure 2. In all 38 cases, host-guest inclusion was observed. In the majority of these 38 pair combinations, only 1:1 host:guest complexes were observed (32 out of 38 cases). Only in a total of six cases were 2:1 host:guest complexes observed, four of them for β-CD (namely *m*-CH_3_, *p*-CH_3_, *m*-ethyl and un-substituted PAN, two of which are shown in Figure 2) and two for γ-CD (namely *m*-CH_3_ and *p*-CH_3_). This lack of higher-order complexes in all but six CD:PAN pairs was reproducible and clearly established by these experiments.

In all six cases in which 2:1 host:guest inclusion complexes were observed, 1:1 complexes were also observed to varying relative concentrations. This indicates a stepwise equilibrium formation of these 2:1 complexes, as follows:

CD + PAN ⇆ {CD:PAN}
(1)

CD + {CD:PAN} ⇆ {CD_2_:PAN} (2)

with equilibrium binding constants K_1_ and K_2_ for Equilibria 1 and 2, respectively. The magnitude of the binding (formation) constants K_1_ and K_2_ cannot be determined from ESI-MS experiments, only the presence (or absence) of these complexes.

**Figure 3:**
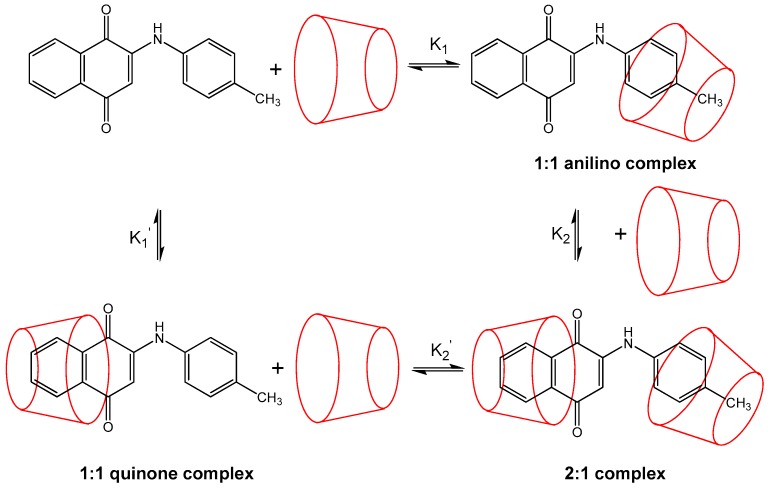
Illustration of the full proposed mechanism for the reversible stepwise formation of the 1:1 and 2:1 host:guest inclusion complexes between β-CD and 2-[(*p*-methyl-phenyl)amine]-1,4-naphthalenedione (PAN derivative 10) observed in the ESI-MS spectrum shown in Figure 2.

The ESI-MS experiments provide direct and unequivocal determination of not only the formation of inclusion complexes but also the varying host:guest ratios (stoichiometries) observed. However, no experimental information is provided by this technique on the nature of these complexes (nor the magnitude of the binding constants, as discussed above), for example the specific orientation in which all or part of the PAN guest fits inside the CD cavity. In the case of PAN, as shown in Figure 1, it is possible to form 1:1 complexes by inclusion by the CD of either the anilino or quinone moiety ends. Complexation of either of these anilino or quinine complexes by a second host CD would then result in the same 2:1 complex formed. This results in a somewhat complex mechanism for the stepwise formation of the 2:1 complexes from initial 1:1 complexes as proposed above, due to the possibility of two different 1:1 intermediate complexes. This full set of equilibria is illustrated in Figure 3; in this case two different 1:1 binding constants, K_1_ and K_1_’ (as indicated by Equilibrium 1 above), are needed, for the formation of anilino or quinone complexes, respectively, and also two pairs of 2:1 binding constants, K_2_ and K_2_’ (as indicted by Equilibrium 2), for the addition of the second host CD to either the anilino or quinone 1:1 complexes, respectively.

Considering the shape of the PAN guests, as shown in Figure 1, it is most likely that the phenyl ring of the aniline moiety is being included in the 1:1 complexes, due to the large steric hindrance caused by the two carbonyls on the quinone moiety. In other words, K_1_ is expected to be larger than K_1_’. This preference of inclusion of the anilino moiety over the quinone end is also supported by our molecular mechanics calculations, as will be discussed in Section 2.2.

It is observed that the formation of 2:1 complexes is more likely with β-CD (occurs with four PAN derivative guests) than with γ-CD (occurs only with two PAN derivative guests). This makes sense, considering the relative size of these two hosts: γ-CD is much larger than β-CD, so there is more likely to be unfavourable steric interaction between the two hosts in the case of γ-CD, making the encapsulation of the guest by a second host less likely. Furthermore, the cavity of γ-CD is larger than that of β-CD, making it more likely that the guest can be completely encapsulated within a single host cavity. The reason why only certain guests are encapsulated by β- and γ-CD will be discussed in Section 3, by considering the electronic properties of these different guests.

### 2.2. Molecular Modeling Studies

Molecular modeling calculations were performed for all 19 PAN derivatives as guests encapsulated in the β-CD, to estimate the stabilization energy obtained by complexation (which provides an indication of the relative binding constants). Both modes of encapsulation for 1:1 inclusion were examined and compared, i.e., encapsulation of the anilino moiety (anilino complex) vs. encapsulation of the quinone moiety (quinone complex). Table 2 lists the results of the molecular modeling calculations for the stability of each of the two types of 1:1 complex with β-CD for each of the 19 guests, in terms of ΔE, the difference in energy between the complex and the free host and guest. Also listed is the value of ΔE for the formation of the 2:1 complex, in which *both* ends of the guest PAN are encapsulated by a β-CD host.

As can be seen from Table 2, in most cases there is a greater stabilization (more negative ΔE) for anilino end encapsulation than for quinone end encapsulation, namely in 14 of the 19 cases (only PAN guests 1, 2, 3 and 4 showed a lower energy for the quinone mode of encapsulation, while PAN 16 showed identical energy for the two modes). This provides support for our proposal in Section 2.1 that the 1:1 complexes most likely involve inclusion of the anilino end of the PAN guest.

**Figure 4:**
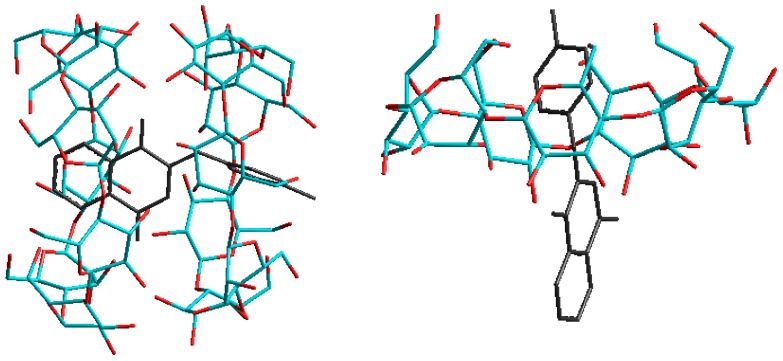
Molecular modeling predicted energy structures for the 2:1 (**left**) and 1:1 anilino (**right**) host:guest complexes formed between β-CD and 2-[(*p*-methyl-phenyl)amine]-1,4-naphthalenedione (PAN derivative 1) and observed in the ESI-MS spectrum shown in Figure 2.

Since only PAN derivatives 1, 4, 7 and 10 showed a 2:1 complex under ESI conditions, we further investigated the results indicated in Table 2 to understand why only 1:1 complexes were present for the other PAN derivatives. The PAN derivatives 1, 4, 7 and 10 contain a relatively small substituent (H, *m*-Et, *m*-Me and *p*-Me respectively) on the aniline end of the molecule. Those four substituents being very small, they have little steric effect on the stability of the complexation. They also do not cause strong electronic effects, since they are only very weakly electron donating (the electronic effects will be discussed in Section 3). Since no considerable steric or electronic effects are present, these 2:1 complexes are stable and are observed in the ESI spectra (except for derivative 9 with a *p*-Et group). Figure 4 shows the structures calculated via molecular modeling for the 2:1 as well as the 1:1 anilino complex of β-CD with PAN derivative 10 (*p*-methyl). This figure illlustrates the minimal steric interaction between the CD cavity and the small *p*-methyl substituent on the anilino ring, and the deep anilino penetration into the CD cavity, in both cases.

PAN derivatives 5 and 6 (*p*-n-Bu and *p*-n-Hexyl respectively) did not show a 2:1 complex since the *n*-butyl and *n*-hexyl groups are quite bulky and under the energy of the ESI, the complexation on the aniline side of the molecule would be lost.

For the other PAN derivatives that did not show a 2:1 complex in ESI, we examined the electronic effects of these substituents since none of them is really bulky enough to cause a significant steric effect. For all the other PAN derivatives (2, 11, 15 and 18) with *meta* substituents (*m*-NO_2_, *m*-CO_2_H, *m*-Cl, *m*-F), the energy difference between the 1:1 quinone and 1:1 anilino complexes are relatively identical (see Table 2). In this case, under ESI energy if one of the β-CD is lost it is difficult to determine which of the two complexations will be lost.

This leaves PAN derivatives 3, 8, 12, 13, 14, 16, 17, and 19. All of these derivatives have a *para* substituent. Comparing derivatives 16 and 18 (*p*-Cl and *p*-Br respectively) shows that the 1:1 anilino complexation for *p*-Br (derivative 17) is favored. This is due to the inductive effect of the *p*-Br which forces the nitrogen atom of the aniline group to push its electronic density into the aromatic. This further stabilizes the aniline moiety versus the quinone. Derivatives 8, 12, 13, 14 and 19 (*p*-NO_2_, *p*-CO_2_H, *p*-CN, *p*-CF_3_ and *p*-COCH_3_), all show the same trend since these groups are electron withdrawing and also force the nitrogen atom to donate its electronic density to the aniline side of the molecule. Derivative 3 (*p*-OMe) shows the exact opposite effect since it is an electron donating group. The electronic density of this group forces the nitrogen atom to push its electronic density into the quinone group. In this case, the 1:1 quinone complexation will be favored.

Thus, when a derivative does not show a 2:1 complexation in the ESI, the absence of these 2:1 complex are explained by a combination steric and electronic effects. Furthermore, the preference for either the 1:1 quinone or 1:1 anilino is correlated by the difference of energy from Table 2.

## 3. Discussion

From the molecular modeling studies described above, it is clear that the electron withdrawing/donating abilities of the various substituents on this set of guests plays a significant role in the nature of the inclusion complexes obtained. This effect can of course be investigated quantitatively using the well-known set of Hammett σ_m_ and σ_p_ substituent constants, which have been determined and tabulated based on the effect of *meta* or *para* substitution of a substituent the acid equilibrium constant of benzoic acid [45,46]. These parameters take into account the inductive and resonance effect of the substituent on the electron distribution of the aromatic ring(s). In general, substituents can be considered as either electron-donating groups (increased aromatic electron density) or electron-withdrawing groups (decreased aromatic electron density). Electron-donating groups have negative σ_m_ and σ_p_ substituent constant values, whereas electron-withdrawing groups have positive σ_m_ and σ_p_ substituent constant values.

It is clear from Table 3 that, other than the unsubstituted PAN itself, only PAN derivatives with substituents with negative σ_m_ values, i.e., with electron donating groups, exhibit 2:1 CD:PAN complexes with β-CD in the ESI-MS spectrum. No PAN derivative with a *meta* substituent with a positive σ_m_ forms 2:1 complexes with β-CD. A similar result is observed for *para*-substituted PAN derivatives with β-CD, as shown in Table 4: the only ones that form 2:1 complexes are those with substituents with negative σ_p_ values, i.e., with electron donating groups. Once again, no PAN derivative with a *para* substituent with a positive σ_p_ forms 2:1 complexes with β-CD. Thus, a relatively high aromatic electron density is required for the formation of 2:1 complexes, which is supported by the presence of electron-donating substituents. However, not all derivatives with negative σ_m_ *meta* substituents show 2:1 complexes: ethyl, n-butyl, and n-hexyl all have negative values but do not form 2:1 complexes. In the latter two cases, the steric effect of these larger alkyl substituents must play a role. In the case of ethyl substitution, the steric effect is seen to be intermediate between the small methyl group and the larger n-butyl, and n-hexyl groups: ethyl does form 2:1 complexes with β-CD when substituted in the *meta* position, but not in the *para*. It is interesting and informative to note that the only substituent which gave 2:1 complexes in all four cases (*meta* and *para* substitution, β- and γ-CD) was the smallest alkyl substituent, the methyl group.

Table 5 and Table 6 show the analogous results in the case of the larger γ-CD; in this case, only methyl substituents show 2:1 complexes. As discussed in Section 2.1 above, it is less likely that 2:1 host:guest complexes will form with the much larger γ-CD, as there would be much more steric interference between the two hosts encapsulating the same PAN guest. In this case, the electron donating requirement of the guest is more important, to help overcome the host steric effect, as evidenced by the fact that the unsubstituted PAN does not form 2:1 complexes (contrary to the result in β-CD). It is, however, surprising that the larger alkyl substituents do not encourage 2:1 complexation, as a larger guest would be more likely to be encapsulated by two hosts.

**Figure 5:**
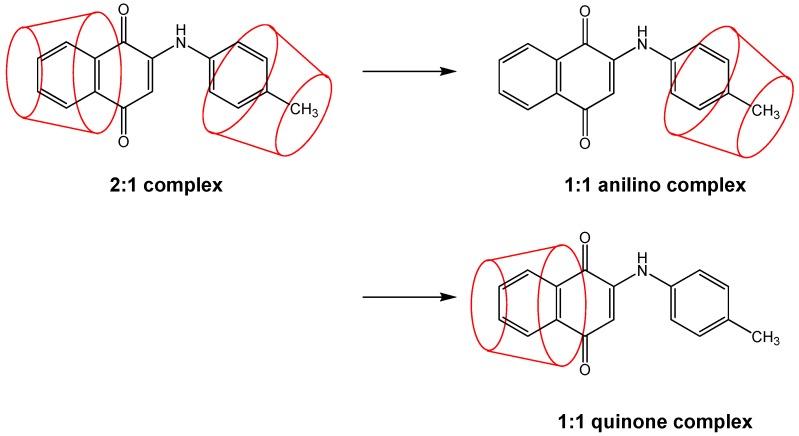
Hypothesis for the lack of observed 2:1 complexes in the case of most PAN derivatives: loss of one CD host to form 1:1 complexes, either anilino or quinone (consistent with the overall scheme shown in Figure 3).

It should be mentioned that in the ESI-MS experiments, only complexes which survive the ionization process are observed. Thus, there is a small possibility that 2:1 complexes form in most cases (especially considering the experimental conditions of a large excess of cyclodextrin), but that such complexes fall apart under MS-ESI conditions for all but a few of the PAN derivatives, resulting in only 1:1 complexes, either anilino (most likely), quinone, or both, being observed in the ESI experiments. This possibility is depicted in Figure 5.

## 4. Materials and Methods

PAN derivatives **1**–**19** were synthesized as previously described [28]. β- and γ-CD were obtained from Sigma-Aldrich (St. Louis, MO, USA), and used as received.

Electrospray ionization mass spectroscopy (ESI-MS) experiments were performed on a Quattro II Micromass (Waters, Elstree, Hertfordshire, UK) spectrometer (CEN de Saclay, Gif-sur-Yvette, France). Solutions contained 10^−6^ to 10^−7^ M of the PAN derivative, with a large (three- to ten-fold) excess of cyclodextrin). Samples were injected at 10 mL/min, with a source temperature of 80 °C and capillary voltage maintained at +3.35 KV. The cone voltage ranged from 10–70 V; at 20 V the skimmer voltage was 1.9 V. In a typical experimental setting, 100 μL of an ethanol solution of the CD and PAN (10:1 mole ratio; large excess of CD used to allow for formation of higher-order host:guest complexes ) was prepared, in some cases a drop of formic acid was added, and introduced through a Harvard Apparatus syringe pump. The ions were detected by scanning the first quadrupole and the mass range was monitored from *m*/*z* = 80–2000 in 7 s. At least 50 scans were averaged to obtain representative spectra.

Molecular modeling calculations were performed on HyperChem 6 MM+ (U de M) in gas phase and in solvent box (ethanol) modes. The minimization of energy was performed using the technique described in the HyperChem instruction manual, and in references [39] and [41]. Energies obtained were minimized potential energies as defined in the HyperChem 6 MM+ approach, and include conformational motions for the obtaining the lowest energy complex configuration. These calculations were done in the same way across the entire set of host:guest pairs, allowing for relatiave stability comparisons to be made. In a first phase, 1600 Polak Ribiere iterations were applied. A small incremental energy was applied in a second phase (0.001 ps for a total of 1 ps, 370 K) for selected insertion models only. All calculated complex structures (1:1, 2:1 and 1:2 stoichiometry) and energy data are available upon request from C.K. Jankowski (U de M).

## 5. Conclusions

2-[(R-phenyl)amine]-1,4-naphthoquinones (PAN) form host-guest inclusion complexes with both β- and γ-CD, as observed by ESI-MS experiments. For the majority of the 19 PAN derivatives studied in these two CDs, only 1:1 CD:PAN complexes were observed (32 out of 38 host:guest pairs). In six cases, 2:1 host:guest complexes were also observed: 4 cases for β-CD (parent PAN, *m*-methyl, *m*-ethyl, and *p*-methyl) and 2 cases for the larger γ-CD (*m*- and *p*-methyl). Thus, the specific nature of the PAN derivative, and the effect of the specific substituents, has a major effect on determining the resulting host-guest stoichiometry, as does the size of the host CD cavity. Molecular modeling studies showed that 1:1 complexation most likely involves encapsulation of the aniline rather than the quinone moiety, and indicated large stabilization upon 2:1 complexation. Consideration of the Hammett parameters of the substituents showed that 2:1 host-guest complexes formed only with PAN derivatives with small substituents with negative σ_m_ or σ_p_ values, i.e., for guests with electron donating substituents. This trend indicates that higher electron density on the aromatic ring encourages complexation by 2 host molecules. However, larger alkyl substituents with negative σ_m_ and σ_p_ parameters did not yield 2:1 complexes, even for the larger γ-CD host, showing that steric effects in terms of guest size are also highly important. It is clear that the prediction of host-guest stoichiometry for a specific host-guest pair is complicated, and involves a subtle interplay of both electronic and steric factors. However, as demonstrated in this paper, there are trends in terms of the electronic nature of the guest and the size of both the guest and host, which can be used to help predict the expected host:guest stoichiometry for a given host-guest pair.

## Figures and Tables

**Table 1:** Substituents and substitution pattern for 2-[(R-phenyl)amine]-1,4-naphthalenedione (PAN) derivatives **1**–**19**, and their CD:PAN complex stoichiometries as observed by MS-ESI spectrometry.

Guest	R_1_ (*meta*)	R_2_ (*para*)	1:1 β-CD	2:1 β-CD	1:1 γ-CD	2:1 γ-CD
**1**	H	H	✓	✓	✓	✕
**2**	NO_2_	H	✓	✕	✓	✕
**3**	H	OMe	✓	✕	✓	✕
**4**	Et	H	✓	✓	✓	✕
**5**	H	^n^Bu	✓	✕	✓	✕
**6**	H	*n*-hexyl	✓	✕	✓	✕
**7**	Me	H	✓	✓	✓	✓
**8**	H	NO_2_	✓	✕	✓	✕
**9**	H	Et	✓	✕	✓	✕
**10**	H	Me	✓	✓	✓	✓
**11**	CO_2_H	H	✓	✕	✓	✕
**12**	H	CO_2_H	✓	✕	✓	✕
**13**	H	CN	✓	✕	✓	✕
**14**	H	CF_3_	✓	✕	✓	✕
**15**	Cl	H	✓	✕	✓	✕
**16**	H	Cl	✓	✕	✓	✕
**17**	H	Br	✓	✕	✓	✕
**18**	F	H	✓	✕	✓	✕
**19**	H	COCH_3_	✓	✕	✓	✕

**Table 2:** Molecular modeling calculations for the energy stabilization upon complexation (ΔE, in kcal/mol) for the inclusion of the 19 PAN derivatives, as 1:1 (anilino encapsulation), 1:1 (quinone encapsulation), or 2:1 (both ends encapsulated) complexes.

Guest	R_1_ (*meta*)	R_2_ (*para*)	ΔE 1:1 (Anilino)	ΔE 1:1 (Quinone)	ΔE 2:1 (Both Ends)
1	H	H	−19.82	−21.77	−31.37
2	NO_2_	H	−20.95	−21.51	−29.69
3	H	OMe	−19.23	−25.16	−37.79
4	Et	H	−20.99	−22.80	−33.73
5	H	^n^Bu	−30.04	−21.51	−48.26
6	H	n-hexyl	−30.92	−24.44	−52.03
7	Me	H	−31.09	−28.71	−40.63
8	H	NO_2_	−23.44	−19.06	−34.86
9	H	Et	−22.61	−20.61	−49.07
10	H	Me	−21.76	−20.01	−48.72
11	CO_2_H	H	−23.07	−22.41	−31.10
12	H	CO_2_H	−30.22	−25.15	−42.80
13	H	CN	−24.3	−16.43	−39.65
14	H	CF_3_	−19.52	−17.64	−36.38
15	Cl	H	−30.18	−27.45	−38.21
16	H	Cl	−22.27	−22.27	−27.92
17	H	Br	−30.43	−28.66	−54.34
18	F	H	−27.23	−27.94	−44.17
19	H	COCH_3_	−23.61	−22.16	−34.70

**Table 3:** Comparison of *meta*-substituted 2-[(R-Phenyl)amine]-1,4-naphthalenediones which form 2:1 and 1:1 vs. only 1:1 complexes with β-CD.

Guest	Stoichiometry	R_1_ (*meta*)	σ_m_ (R_1_)
1	1:1 and 2:1	H	0
4	1:1 and 2:1	Et	−0.07
7	1:1 and 2:1	Me	−0.06
2	1:1 only	NO_2_	0.72
11	1:1 only	CO_2_H	0.35
15	1:1 only	Cl	0.37
18	1:1 only	F	0.34

**Table 4:** Comparison of *para*-substituted 2-[(R-Phenyl)amine]-1,4-naphthalenediones which show 2:1 and 1:1 vs. only 1:1 complexes with β-CD.

Guest	Stoichiometry	R_2_ (*para*)	σ_p_ (R_2_)
1	1:1 and 2:1	H	0
10	1:1 and 2:1	Me	−0.14
3	1:1 only	OMe	*−*0.27
5	1:1 only	^n^Bu	*−*0.16
6	1:1 only	n-hexyl	*−*0.16
8	1:1 only	NO_2_	0.78
9	1:1 only	Et	*−*0.15
12	1:1 only	CO_2_H	0.44
13	1:1 only	CN	0.66
14	1:1 only	CF_3_	0.53
16	1:1 only	Cl	0.23
17	1:1 only	Br	0.23
19	1:1 only	COCH_3_	0.50

**Table 5:** Comparison of *meta*-substituted 2-[(R-Phenyl)amine]-1,4-naphthalenediones which form 2:1 and 1:1 vs. only 1:1 complexes with γ-CD.

Guest	Stoichiometry	R_1_ (*meta*)	σ_m_ (R_1_)
7	1:1 and 2:1	Me	−0.06
1	1:1 only	H	0
4	1:1 only	Et	−0.07
2	1:1 only	NO_2_	0.72
11	1:1 only	CO_2_H	0.35
15	1:1 only	Cl	0.37
18	1:1 only	F	0.34

**Table 6:** Comparison of *para*-substituted 2-[(R-Phenyl)amine]-1,4-naphthalenediones which form 2:1 and 1:1 vs. only 1:1 complexes with γ-CD.

Guest	Stoichiometry	R_2_ (*para*)	σ_p_ (R_2_)
10	1:1 and 2:1	Me	−0.14
1	1:1 only	H	0
3	1:1 only	OMe	−0.27
5	1:1 only	^n^Bu	−0.16
6	1:1 only	n-hexyl	−0.16
8	1:1 only	NO_2_	0.78
9	1:1 only	Et	−0.15
12	1:1 only	CO_2_H	0.44
13	1:1 only	CN	0.66
14	1:1 only	CF_3_	0.53
16	1:1 only	Cl	0.23
17	1:1 only	Br	0.23
19	1:1 only	COCH_3_	0.50
